# Evaluating clinical utility of subgingival and salivary endotoxin activity levels as periodontal biomarkers

**DOI:** 10.3389/froh.2022.1029806

**Published:** 2022-11-01

**Authors:** Svetislav Zaric, Alexander Strachan, Yuko Kurushima, Anbo Dong, Clare McIlwaine, Zoe Harrington, Luigi Nibali, Andrew Foey, Mark Ide

**Affiliations:** ^1^ Centre for Host-Microbiome Interactions, Faculty of Dentistry, Oral & Craniofacial Sciences, King's College London, London, United Kingdom; ^2^ Faculty of Health, University of Plymouth, Plymouth, United Kingdom; ^3^ Eastman Dental Hospital, University College London Hospital NHS Trust, London, United Kingdom

**Keywords:** periodontitis, biomarkers, LPS, endotoxin, saliva, plaque

## Abstract

**Objectives:**

The use of periodontal biomarkers for identification and monitoring of unique patient populations could foster better stratification of at-risk groups, increase access to treatment for those most in need, facilitate preventive measures and improve personalised care plans. The aim of this study was to examine the diagnostic and prognostic utility of oral lipopolysaccharides as bacterially-derived periodontal biomarkers.

**Methods:**

Periodontal parameters were recorded, and saliva and subgingival plaque samples were collected at the beginning of the study from periodontally healthy volunteers and periodontitis patients, and three months after completion of conventional periodontal treatment in the periodontitis group. Endotoxin activity in the samples was measured using the recombinant factor C assay. Associations between clinical periodontal parameters and subgingival and salivary endotoxin activities were analysed using a multivariate regression model, while the ROC curve was applied to estimate the sensitivity, specificity and c-statistics for salivary and subgingival endotoxin activities as diagnostic biomarkers for periodontitis.

**Results:**

Significant correlations were found between subgingival endotoxin activities, probing pocket depth and periodontal diagnosis, which were independent from patients' age, gender and smoking status. In addition, subgingival endotoxin levels had high specificity and sensitivity in detecting periodontal health and disease (0.91 and 0.85 respectively). Salivary endotoxin activity was positively associated with periodontal diagnosis, mean probing pocket depth, percentages of sites over 4 mm and full mouth bleeding score. However, it was inferior in discriminating patients with stable periodontium from those with periodontitis (sensitivity = 0.69, specificity = 0.61) compared to subgingival endotoxin activity.

**Conclusions:**

Subgingival endotoxin activity has good diagnostic and prognostic values as a site-specific periodontal biomarker and is not influenced by the patient's age, gender or smoking status. In contrast, salivary endotoxin activity, as a patient-level biomarker, is dependent on patient's age, has poorer diagnostic and prognostic capability, but shows good correlations with disease susceptibility and both its extent and severity.

## Introduction

Personalised approaches for disease prevention and treatment aim to determine the predisposing factors that can lead to disease and deliver timely and targeted prevention and treatment measures. Understanding the risk factors that trigger the health-to-disease transition in periodontal tissues is essential for delivering personalised prevention measures and reducing the burden of this chronic inflammatory disease (1).

Diagnostic methods in today's clinical periodontal practice lack the ability to objectively predict the onset of the disease or to identify those patients at higher risk for future periodontal tissues breakdown and tooth loss. The potential use of periodontal biomarkers for identification and monitoring of unique patient populations could foster better stratification of at-risk groups, increase access to treatment for those most in need, facilitate preventive measures and improve personalised care plans (2). Measuring the risk of periodontal disease progression at the individual and tooth levels can be highly informative for planning personalised, risk-based periodontal care.

Periodontal infection is initiated by tooth-associated microbial biofilms that stimulate a host inflammatory response, leading to soft tissue destruction and alveolar bone loss. The relationship between the oral microbiome and the host's immuno-inflammatory response in the pathogenesis of periodontal diseases is complex. Periodontal diseases are caused by synergistic and dysbiotic oral microbial communities rather than by specific periodontopathogens. In this polymicrobial synergy, different community members and their virulence factors converge together to shape and stabilise a disease-provoking microbiota (3). The shift to this dysbiotic microflora appears to be largely influenced by excessive, persistent inflammation and pocket formation which changes the bacterial growth environment. An important core function for the pathogenicity of this dysbiotic community is the expression of diverse molecules (adhesins, enzymes and proinflammatory ligands), which in combination, act as community virulence factors in order to nutritionally sustain heterotypic and proinflammatory microbial complexes that assist a non-resolving and tissue-destructive host inflammatory response (4).

Bacterial lipopolysaccharides (LPS) have a proinflammatory effect on most of the cell types present in the periodontium, such as macrophages, fibroblasts, lymphocytes, gingival epithelial cells, osteoblasts and osteoclasts (5). LPS is the major constituent of the outer membrane of Gram-negative bacteria, where it plays an important structural role and mediates interaction between bacteria and the environment. Lipid-A, a bioactive centre of LPS, is considered to be an archetypal microbe-associated molecular pattern (MAMP), which the innate immune system recognizes as non-self through an extensive repertoire of evolutionary conserved Pattern Recognition Receptors (PRRs) such as Toll-like Receptors (TLR) and Scavenger Receptors (SRs) (6). Activation of PRRs by lipid-A triggers intracellular signalling cascades that lead to secretion of pro-inflammatory cytokines. TLRs can be activated by structurally diverse lipid-A molecules, and minor changes in the structure of the lipid-As chemical composition can affect their endotoxin activity and inflammatory potential (7).

It has been shown that characteristic lipid A molecular signatures, corresponding to over-acylated, bi-phosphorylated lipid A isoforms, are present in dental plaque and saliva of periodontitis patients. In addition, endotoxin activity levels [measured by the recombinant Factor C (rFC) assay] of subgingival and salivary LPS extracts from periodontitis patients were significantly higher compared to healthy volunteers (8, 9). Monitoring endotoxin activity in oral fluids could be a valuable risk assessment tool for primary and secondary prevention of periodontal diseases and personalised treatment choices, giving an opportunity to intervene closer to the biologic onset of the disease.

A periodontal biomarker can only be used to guide the management of periodontal conditions if it has analytical validity, is accurate, reproducible and reliable, and if it has been shown to have a clinical utility. The aim of this study was to examine the diagnostic and prognostic utility of oral lipopolysaccharides as bacterially-derived periodontal biomarkers by correlating subgingival and salivary endotoxin activity levels with periodontal clinical parameters.

## Materials and methods

### Study design and population

This was a prospective, interventional study performed in a primary dental care setting. Approval of the study protocol was obtained from the Health Research Authority, UK (14/SW/0020). Thirty-two patients (11 female, 21 male, mean age 46) with generalised, unstable periodontitis stages II to IV, and 33 systemically and periodontally healthy persons (18 female, 15 male, mean age 31), with at least 20 teeth were recruited from a primary dental care setting.

The primary outcome for this study was the subgingival and salivary endotoxin activity level at baseline. The sample size calculation was based on data from a previous study that measured salivary endotoxin activity in periodontitis patients (10). From this study, a difference of 2,500 EU/ml (SD = 2,000 EU/ml) in salivary endotoxin activity was detected between healthy and periodontitis patients. At 90% power and a 5% significance level, a sample size of *n* = 28 in each group was required to detect a minimum difference of at least 1 standard deviation of salivary endotoxin activity levels between healthy and disease patients.

Periodontitis patients were diagnosed in accordance with the following clinical criteria: radiographic alveolar bone loss >15% affecting more than 30% of teeth with probing pocket depths ≥5 mm and bleeding on probing (BOP). Periodontal health/stability was defined as probing pocket depths ≤3 mm, no more than 10% BOP and no signs of radiographic bone loss (11). Exclusion criteria included the following: self-reported diagnosis of any systemic illnesses known to affect the periodontal status, regular use of medication to control systemic illness; antibiotic use within 3 months before the beginning of the study, periodontal treatment in the previous 6 months and pregnant or lactating patients.

### Clinical examination, periodontal treatment and samples collection

Full-mouth plaque and bleeding scores were assessed by two aligned (using extra-oral models) examiners and detailed 6-point periodontal charting was recorded using a UNC-15 periodontal probe at six sites per tooth, with measurements rounded to the nearest millimetre. Self-reported smoking status was recorded as “never,” “current” or “previous”.

Unstimulated, whole saliva samples were collected into sterile universal tubes by expectoration for 5 min and not less than 30 min after eating, drinking or smoking. Subgingival plaque samples were collected by inserting sterile, absorbent paper points size 40 (Dentsply) for 30s in three deepest bleeding pockets in periodontitis patients and in healthy patients from three non-bleeding sites. The samples were stored at −80 °C until LPS analyses.

After initial clinical measurement and sample collection, periodontitis patients received oral hygiene instructions, dental health education and a full-mouth supragingival professional, mechanical plaque removal. Root surface instrumentation (RSI) was performed under local anaesthesia using periodontal curettes and ultrasonic devices, in three or four appointments (within two weeks period) without time limitations. RSI was not considered complete until the tooth surface felt smooth on tactile inspection by an explorer. Recruitment of all participants was done within one year period and the same examiners (ZH and CM) were responsible for recruitment, treatment, baseline and follow-up clinical examinations and sampling of respective patients.

Periodontitis patients were recalled three months after the completion of periodontal treatment, clinical parameters were measured and samples (unstimulated mixed saliva and subgingival plaque samples from the same sites) collected as per baseline visit.

### LPS extraction and assessment of endotoxin activity

LPS from salivary and subgingival plaque samples was extracted using the LPS extraction kit (iNtRON Biotechnology, S. Korea), following the manufacturer's instructions. Extracted LPS was re-suspended in 500 µl of LPS-free water and stored at 4 °C. Endotoxin activity of salivary and subgingival LPS extracts was measured (EU/ml), in duplicates, by an endpoint, fluorescent, recombinant Factor C assay according to manufacturer's instructions (EndoZyme, Hyglos, Germany). All samples were anonymised and laboratory staff were blinded until the end of the study.

### Statistical analyses

Associations between clinical periodontal parameters and subgingival and salivary endotoxin activities, adjusting for participants' age, smoking status and gender were analysed using a multivariate regression model. The correlations between salivary/subgingival endotoxin activities and clinical parameters was examined by Spearmans' rank correlation test. Wilcoxon Test was used for comparing the pre- and post-treatment data in periodontitis patients.

For the periodontal site-level analyses, the mean probing pocket depth of the three sampled periodontal sites was used as the main clinical parameter. For the person-level analyses, full-mouth probing pocket depth (PPD) and clinical attachment level (CAL) means, percentage of pockets ≥4 mm as well as the plaque to bleeding ratio (FMPS/FMBS) were calculated.

The receiver operating characteristic (ROC) curve was applied to estimate the sensitivity, specificity and c-statistics (area under the curve: AUC) for salivary/subgingival endotoxin activity as diagnostic biomarkers for periodontitis.

Multivariate regression analyses were performed with the GEE package, version 4.13–20. Statistical significance was set at *p*-value < 0.05. Data were analysed using the statistical software R version 4.0.3.

## Results

### Demographic, clinical and biological characteristics of the study population

Periodontitis patients (age range 39–67) were significantly older than healthy volunteers, but overall, the study population belonged to the middle-aged group as the mean age was 46. Smokers and ex-smokers were more prevalent in the periodontitis group.

All baseline clinical characteristics were significantly worse in the periodontitis group, whilst there were significant improvements in the full mouth plaque and bleeding scores and probing pocket depths following periodontal treatment. Subgingival endotoxin activity was significantly elevated in diseased sites compared to healthy sites and returned to almost healthy levels after periodontal treatment. In contrast, salivary endotoxin activity was significantly higher in periodontitis patients compared to healthy persons and sustained at high levels even after periodontal therapy (Table 1).

**Table 1:** Demographic, clinical and endotoxin activity characteristics (with SDs) of the study population with differences between pre- and post-treatment in the periodontitis group. Endotoxin activity (EA) expressed in endotoxin units per ml (EU/ml).

	Healthy (*N* = 33)	Periodontitis (*N* = 32)	
Age	31 ± 9	47 ± 8	
Gender (*N*)			
Male	13	21	
Female	20	11	
Smoking status (*N*)			
Never	28	7	
Previous	2	11	
Current	3	14	
		**Pre-treatment**	**Post-treatment**
FMPS (%)	21.05 ± 16.87	55.55 ± 20.77	36.4 ± 16.91
FMBS (%)	2.07 ± 3.32	37.99 ± 19.37	23.73 ± 16.75
FMPS/FMBS	15.81 ± 15.58	3.74 ± 10.57	5.33 ± 12.11
Mean Pocket depth (mm)	1.46 ± 0.45	3.54 ± 0.61	2.8 ± 0.57
Mean CAL (mm)	1.39 ± 0.38	4.32 ± 0.99	3.55 ± 0.86
Mean Recession (mm)	0.04 ± 0.08	0.78 ± 0.77	1.25 ± 0.69
Mean site depth (mm)	1.25 ± 0.4	6.01 ± 1.14	4.79 ± 1.2
Percentage of sites >4 mm pocket depth	0.32 ± 0.76	42.73 ± 19.21	22.3 ± 15.52
Salivary EA (EU/ml)	102.12 ± 183.53	313.9 ± 371.88	334.6 ± 296.25
Subgingival EA (EU/ml)	5.97 ± 16.23	71.99 ± 89.55	16.19 ± 21.73

### Association between subgingival endotoxin activity and clinical parameters

**Figure 1:**
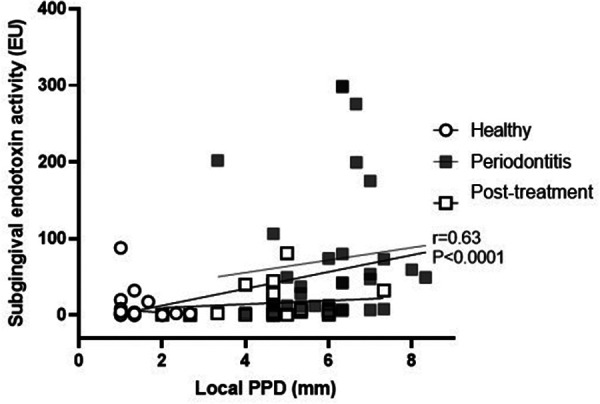
Association between subgingival endotoxin activity and the local probing pocket depth.

Subgingival endotoxin activity was significantly associated with the local probing pocket depth (correlation coefficient 0.63; 95% CI: 0.48, 0.75; *p* < 0.0001) (Figure 1). This correlation remained statistically significant even when adjusted for gender, age and smoking status (*p* = 0.001) (Table 2).

**Table 2:** Multivariate analysis of association between subgingival endotoxin activity levels and site probing pocket depth, adjusted for age, gender and smoking status.

	Coefficient (95% CI)	*p*-value
SITE DEPTH	14.72 (6.27, 23.16)	0.0012
Age	−1.21 (−2.89, 0.47)	0.16
Gender (Male)	−2.71 (−34.04, 28.62)	0.87
Smoking (Former)	40.26 (−7.40, 87.93)	0.10
Smoking (Current)	−4.02 (−47.03, 38.98)	0.86

In addition, subgingival endotoxin activity from the three deepest periodontal pockets was significantly associated with the overall periodontal status / diagnosis (*p* = 0.00047) and this association was also independent from the patients' age, gender and smoking habits (Table 3).

**Table 3:** Multivariate analysis of association between subgingival endotoxin activity and periodontal diagnosis, adjusted for age, gender and smoking status.

	Coefficient (95% CI)	*p*-value
Periodontal status	88.29 (41.54, 135.05)	0.00047
Age	−1.57 (−3.30, 0.16)	0.08
Gender (Male)	−9.33 (−40.83, 22.17)	0.56
Smoking (Former)	35.29 (−12.27, 82.85)	0.15
Smoking (Current)	−13.36 (−57.23, 30.51)	0.55

**Figure 2:**
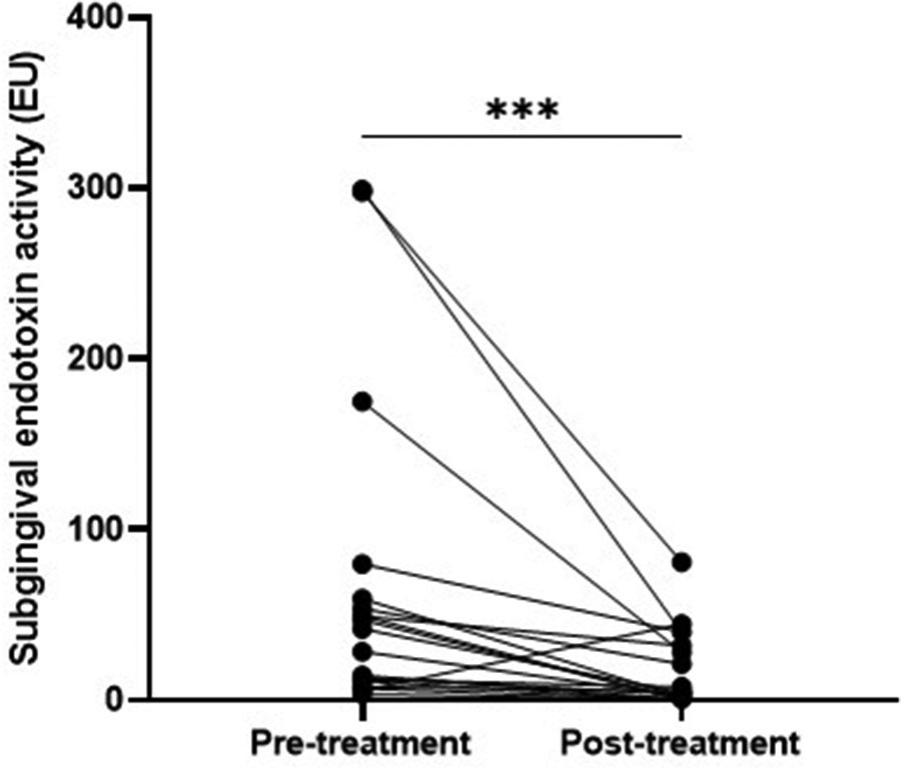
Comparison of subgingival endotoxin activities (EU/ml) in periodontitis patients before and after periodontal treatment.

All periodontitis patients, apart from one, had significantly lower levels of subgingival endotoxin activity after the periodontal treatment (Wilcoxon matched-pairs *p* = 0.0003) (Figure 2).

### Association between salivary endotoxin activity and clinical parameters

**Figure 3:**
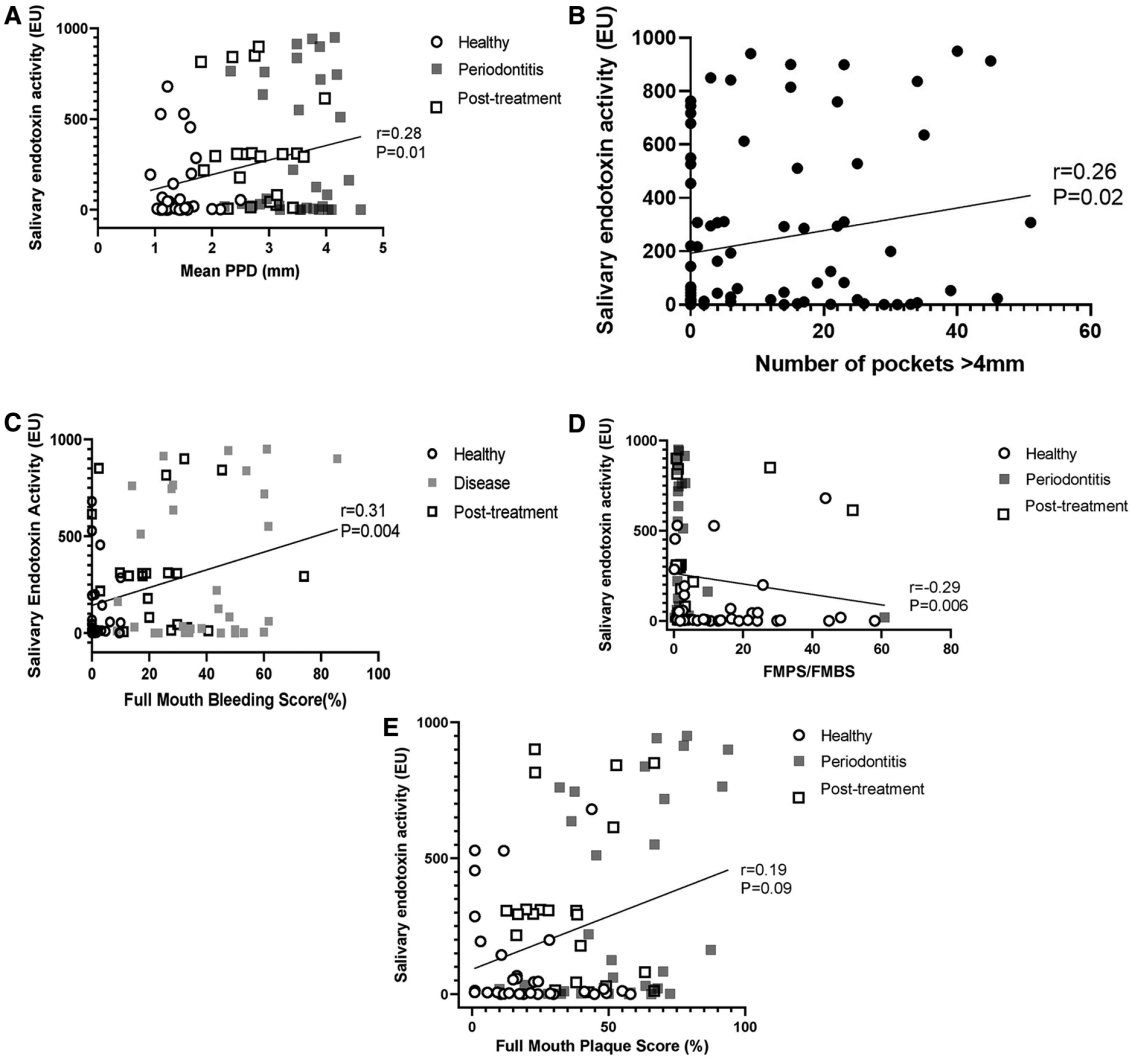
(**A**) correlation between salivary endotoxin activity and full mouth mean probing pocket depth. (**B**) Correlation between salivary endotoxin activity and number of sites with PPD > 4 mm. (**C**) Correlation between full mouth bleeding score and salivary endotoxin activity. (**D**) Salivary endotoxin activity is inversely correlated with the FMPS/FMBS ratio. (**E**) Salivary endotoxin activity is not correlated with the full mouth plaque scores.

Salivary endotoxin activity was positively associated with periodontal diagnosis (health/periodontitis) (*p* = 0.006), mean probing pocket depth (Correlation coefficient: 0.28; 95% CI: 0.06, 0.47; *p* = 0.01) (Figure 3A), number of pockets over 4 mm (Correlation coefficient: 0.26; 95% CI: 0.05, 0.46; *p* = 0.02) (Figure 3A) and full mouth bleeding score (Correlation coefficient = 0.31; 95% CI: 0.09, 0.49), *p* = 0.004) (Figure 3C). Interestingly, salivary endotoxin levels were negatively associated with the FMPS/FMBS ratio (Correlation coefficient: −0.29; 95%CI: −0.48, −0.08; *p* = 0.006) (Figure 3D), while the correlation with the full mouth plaque score was not statistically significant (r = 0.19, 95% CI: −0.03, 0.39: *p* = 0.09) (Figure 3E).

However, after adjusting these univariate correlations between salivary endotoxin activity and periodontal clinical parameters for age, gender and smoking status, patient's age was the most important factor influencing these relations, while smoking has a tendency to decrease salivary endotoxin levels (Table 4).

**Table 4:** Multivariate analyses of correlations between salivary endotoxin activity levels, clinical and demographic characteristics of the patient cohort, adjusted for age, gender and smoking status.

	Salivary endotoxin activity					
	Coefficient (95% CI)	*p*-value	Coefficient (95% CI)	*p*-value	Coefficient (95% CI)	*p*-value
Mean PD	0.17 (−0.21, 0.55)	0.39				
FMBS			0.01 (−0.001, 0.03)	0.08		
FMPS/FMBS					0.17 (−0.04, 0.38)	0.11
Age	0.05 (0.01, 0.08)	0.006	0.05 (0.02, 0.08)	0.002	0.05 (0.025, 0.082)	0.0005
Gender (Male)	−0.17 (−0.77, 0.42)	0.57	−0.23 (−0.82, 0.35)	0.44	−0.11 (−0.7, 0.47)	0.7
Smoking (Previous)	−0.64 (−1.62, 0.34)	0.21	−0.69 (−1.58, 0.2)	0.13	−0.46 (−1.3, 0.39)	0.29
Smoking (Current)	−0.72 (−1.57, 0.13)	0.10	−0.74 (−1.53, 0.04)	0.07	−0.67 (−1.44, 0.11)	0.1

**Figure 4:**
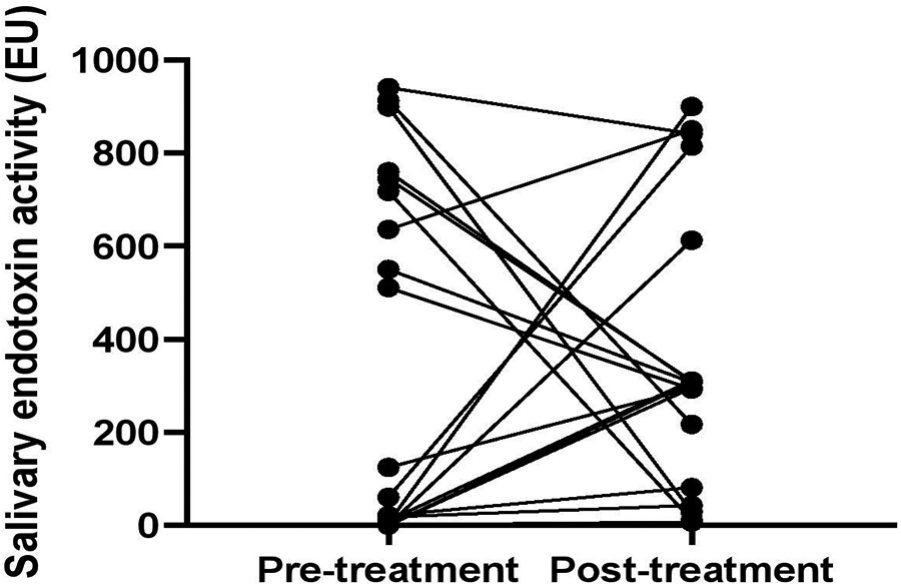
Comparison of salivary endotoxin activity levels in periodontitis patients before and after periodontal treatment.

Overall, there was no a statistically significant difference between the mean salivary endotoxin activity levels in the periodontitis patients group before and after periodontal treatment (*p* = 0.75). In some periodontitis patients the salivary endotoxin activity decreased, while in the others increased after conventional periodontal therapy (Figure 4).

**Figure 5:**
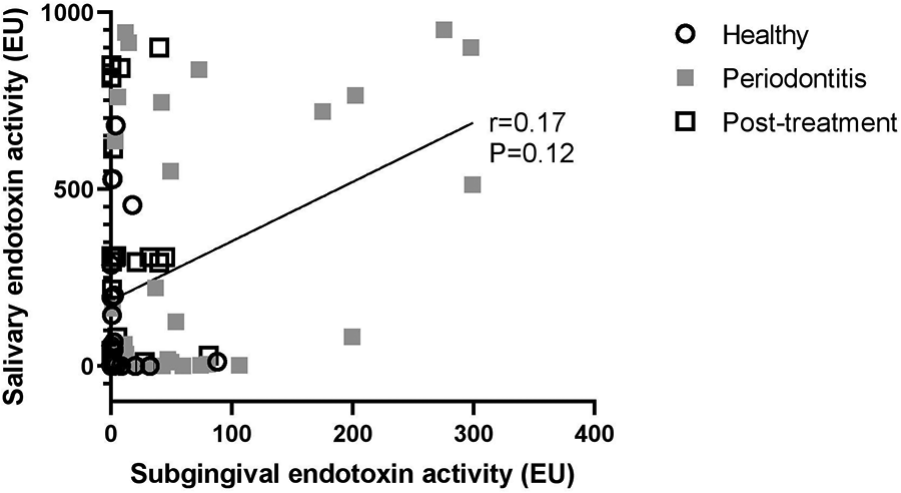
No correlation between subgingival and salivary endotoxin activity levels was detected.

Finally, subgingival endotoxin and salivary endotoxin levels did not correlate with each other in any of the groups of patients (Correlation coefficient: 0.17; 95% CI: −0.06, 0.37; *p* = 0.12 (Figure 5).

### Diagnostic value of subgingival and salivary endotoxin levels

**Figure 6:**
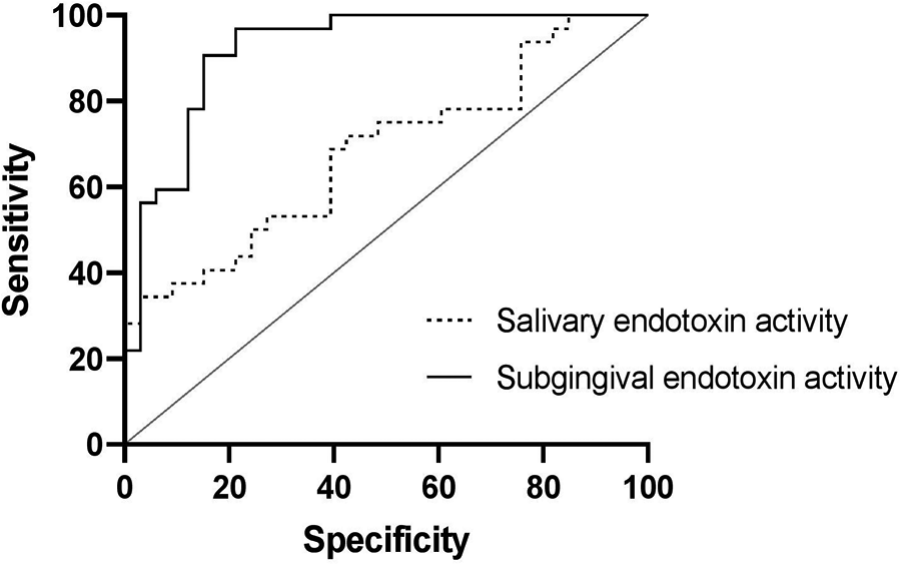
The ROC curves of subgingival endotoxin activity for detection of healthy and diseased sites and salivary endotoxin activity as a periodontal biomarker for detecting periodontal health and disease.

• The receiver operator characteristic (ROC) curves revealed that subgingival endotoxin levels had high specificity and sensitivity in detecting periodontally healthy and diseased sites (0.91 and 0.85 respectively), while salivary endotoxin level was significantly inferior in discriminating patients with stable periodontium from those with periodontitis (sensitivity = 0.69, specificity = 0.61) (Figure 6).

## Discussion

Inflammatory response is a physiological process helping the body to heal against harmful entities, but when dysregulated it could lead to unresolved chronic local or systemic inflammation. The interplay between microbial virulence factors and the person's genotype, phenotype, medical history, nutritional status and life-style stressors is likely to be involved in driving the health-to-disease transition, leading to the onset of chronic diseases (12). Recent developments in personalised periodontics have followed the principles of precision medicine to refine definitions of periodontal health and disease and to identify measurable biologic bases of disease susceptibility, with the ultimate goal of tailoring or targeting preventive and treatment strategies (13).

The three fundamental mechanisms by which measuring a biomarker in the context of clinical care may improve periodontal health are: helping the patient understand their disease or risk of disease; motivating the patient to make behavioural changes that improve periodontal health; and helping a clinician make better informed clinical decisions that lead to improved health of the patient (14).

We evaluated here salivary and subgingival endotoxin activities as potential, bacterially-derived diagnostic and prognostic periodontal biomarkers. Inflammatory potential of bacterial lipopolysaccharides depends on its lipid A chemical composition and can vary from highly TLR agonistic to TLR antagonistic moieties. Endotoxin activity, measured by the recombinant factor C assay, corresponds well with the alterations of lipid A chemical structures and lipopolysaccharide's inflammatory potential (15).

Endotoxin has been associated with dysbiotic subgingival microbial communities and can be successfully removed from the root surfaces by subgingival professional biofilm disruption (16). It has also been shown that gentle mastication is able to induce the release of bacterial endotoxins from the oral origin into the bloodstream, especially when patients have severe periodontal disease, suggesting that a diseased periodontium can be a major and underestimated source of chronic, or even permanent, release of lipopolysaccharide into the bloodstream (17). Patients with severe periodontitis have significantly higher serum endotoxin activity compared to individuals with healthy periodontium and these high levels of activity remained even after periodontal treatment (18), similar to our findings with regards to salivary endotoxin activities. Moreover, subgingival microbial burden contributes to endotoxemia and about 5% of the serum LPS variations could be explainable by salivary LPS among patients with periodontitis (10).

We showed here that subgingival endotoxin levels could be a reliable periodontal biomarker, with high sensitivity and specificity in distinguishing healthy from active periodontal sites. In addition, there was a significant correlation between local probing pocket depth and subgingival endotoxin activity, even when controlling for other analysed patient factors (age, gender, smoking). Moreover, our data implied that subgingival endotoxin activity had good prognostic value, since the periodontal treatment decreased the activity of endotoxin significantly.

In contrast, salivary endotoxin activity levels had a good diagnostic value and they correlated well with the mean probing pocket depth, full mouth bleeding scores, number of pockets over 4 mm and the plaque to bleeding ratio, but had a poor prognostic value as their levels were inconsistent and did not decrease after periodontal treatment, despite the improvements of clinical parameters. In addition, after adjusting for patient's age, the aforementioned correlations were deemed not significant. Interestingly, salivary endotoxin activity did not correlate with the full mouth plaque score, proving that pathogenic potential of plaque does not depend on its amount but rather on the composition (19). The inverse correlation between salivary endotoxin activity and the plaque to bleeding ratio, adds the value to salivary endotoxin activity as a marker for disease risk assessment at a patient level. Previous studies have shown that individuals with a low plaque/bleeding ratio developed significantly more clinical inflammation in terms of bleeding and swelling of the gingiva than individuals with a high plaque/bleeding ratio (20). The inferior clinical utility of salivary endotoxin activity, compared to subgingival LPS activity, could be explained by the contribution of different sources of LPS (from other oral niches) to the total salivary LPS (9). In our study, the levels of salivary endotoxin activities did not show statistically significant correlations with subgingival endotoxin activity, suggesting that subgingival niche might not be the main source of salivary LPS. However, due to very large interindividual variabilities, the preliminary nature of the study and the relatively small sample size, larger study are needed in order to draw definitive conclusions.

The current literature on diagnostic accuracy of single molecular biomarkers in oral fluids features a predominance of individual results from a multitude of mostly host-derived molecules, with MMP8 and elastase being the most researched biomarkers (21). Apart from a rapid, chair-side, salivary detection of *P. gingivalis*, based on monoclonal antibodies to the A1-adhesin domain of the RgpA-Kgp proteinase-adhesin complex (22), our study is one of the few that have evaluated the clinical utility of bacterially-derived periodontal biomarkers. However, the limitations of our study are that it was a preliminary, low-scale study and that the patients were not matched for age. Larger scale studies are needed to confirm these results.

## Conclusions

Subgingival endotoxin activity has good diagnostic and prognostic values as a site-specific periodontal biomarker. It correlates well with the probing pocket depth, significantly decreases after periodontal treatment and is not influenced by the patient's age, gender or smoking status. In contrast, salivary endotoxin activity, as a patient-level biomarker, was dependent on patient's age, had poorer diagnostic and prognostic capability, compared to the subgingival counterpart but did show good correlations with disease extent and severity.

## Data Availability

The raw data supporting the conclusions of this article will be made available by the authors, without undue reservation.
